# Selective Plasticity of Hippocampal Sub‐Regions in the Amnestic Mild Cognitive Impairment After Acupuncture

**DOI:** 10.1002/brb3.70748

**Published:** 2025-08-21

**Authors:** Jinhuan Zhang, Bin Yan, Yongfeng Liu, Ting Liu, Liyu Hu, Xiaohui Zhan, Xinbei Li, Chengcheng Zhu, Jinping Xu, Haibo Yu

**Affiliations:** ^1^ Department of Acupuncture Shenzhen Traditional Chinese Medicine Hospital Shenzhen China; ^2^ Department of Acupuncture The Fourth Clinical Medical College of Guangzhou University of Chinese Medicine Shenzhen China; ^3^ Department of Radiology Traditional Chinese Medicine Hospital Shenzhen China; ^4^ Department of Radiology University of Washington School of Medicine Seattle Washington USA; ^5^ Institute of Biomedical and Health Engineering Shenzhen Institutes of Advanced Technology Chinese Academy of Sciences Shenzhen China

**Keywords:** acupuncture, amnesic mild cognitive impairments, hippocampal subregion, MRI

## Abstract

**Background**: Acupuncture has been demonstrated to be effective in reducing cognitive decline in patients with amnestic mild cognitive impairment (aMCI). Hippocampal atrophy has been typically reported as a core neuromechanism in the aMCI. However, whether and how hippocampal subregions plasticity exists in aMCI after acupuncture remains largely unknown.

**Objectives**: We aimed to explore gray matter alterations in the hippocampal subregions of aMCI after acupuncture.

**Methods**: A randomized, controlled, blind research was conducted. A cohort of 53 patients with aMCI was randomly assigned to either the verum acupuncture (VA) group or the sham acupuncture (SA) group. Each group received 24 treatment sessions (three times per week for 8 weeks). Clinical evaluation and magnetic resonance imaging scans were performed at baseline and after treatment for all patients with aMCI. Hippocampal subfield volumes were analyzed using Freesurfer 7.1.1. An interaction effect was used to explore the efficacy of acupuncture. In addition, a correlation analysis was performed to investigate the relationships between the volume of hippocampal subregions and cognitive function in patients with aMCI.

**Results**: Mixed‐effects analysis revealed a significant group × time interaction for volume in the right subiculum body (*p* = 0.006), where volume significantly decreased following SA (*p* = 0.015) but increased following VA. Within‐group effects showed that VA significantly increased volume in the right GC‐ML‐DG head (*p* = 0.041) and right CA4 body (*p* = 0.028), while SA increased volume in the right CA4‐head (*p* = 0.035). Directly comparing the magnitude of change between interventions, VA led to significantly greater volume increases than SA in the right subiculum body (*p* = 0.008), right CA1‐body (*p* = 0.042), whole hippocampus body (*p* = 0.041), and whole hippocampus (*p* = 0.028). Clinically, within the VA group, the increase in right subiculum body volume was significantly correlated with improvement in AVLT_N5 (delayed recall) scores (*p* = 0.013).

**Conclusion**: These results suggested that acupuncture could selectively induce structural plasticity in hippocampal subregions associated with cognitive effects in patients with aMCI, which provided potential biomarkers—particularly the right subiculum body—for effective and timely interventions for aMCI.

**Trial Registration**: Chinese Clinical Trial Registry (http://www.chictr.org.cn), Registration number: ChiCTR2400084308.

## Graphical abstract

Gay matter alterations in the hippocampal subregions of aMCI after acupuncture. The analysis revealed that patients in aMCI showed increased right subiculum body, right GC‐ML‐DG head, right CA4 body, CA1 body, whole hippocampus body, and whole hippocampus after acupuncture.

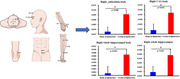

## 1 Introduction

Mild cognitive impairment (MCI) is a condition that occurs as a prior stage of Alzheimer's disease (AD) (Zhang et al. 2015). Amnestic MCI (aMCI), the most common type of MCI, is most likely to progress to AD (Levey et al. 2006) with an annual rate of ∼25% (Petersen et al. 2001). Early aMCI treatment can release cognitive symptoms, to reduce the likelihood of clinical dementia, alleviating the heavy mental and financial burden on society and families. However, pharmacologic treatments for aMCI have proven to be largely unsatisfactory (Petersen et al. 2018), underscoring an urgent need to develop non‐drug treatment options.

Acupuncture, a traditional Chinese medicine technique, is characterized by the insertion of a needle into the skin, followed by manual manipulation or electrical stimulation. It has been used for thousands of years while gaining worldwide attention and recognition (Kim et al. 2019). Numerous previous systematic review suggested that patients with aMCI showed greater clinical efficacy rates, higher mini‐mental state examination (MMSE) scores, and better picture recognition scores after acupuncture (Deng and Wang 2016). In addition, our previous study showed that verum acupuncture (VA) was superior to sham acupuncture (SA) in improving MMSE, Montreal cognitive assessment (MoCA), and auditory verbal learning test (AVLT) (Zhang et al. 2022).

To date, considerable evidence suggests that hippocampal volume loss is a typical biomarker of aMCI susceptibility to conversion to AD (Chen et al. 2020; Zhang et al. 2021; Wei et al. 2023; Ferreira et al. 2011; Zhang et al. 2023; Gerischer et al. 2018). Therefore, the alteration of hippocampal volume is most significant for evaluating treatment efficacy of acupuncture in the aMCI. Indeed, previous neuroimaging studies have demonstrated that acupuncture can lead to functional plasticity of hippocampus in patients with aMCI (Yin et al. 2023; Li et al. 2020). However, evidence about the structural plasticity of hippocampus due to acupuncture for aMCI is limited. Moreover, the hippocampus is not a homogeneous structure; it comprises several subfields with distinct anatomical, functional, and electrophysiological properties (Small et al. 2011; Aggleton 2012). Hippocampal subfields are differentially involved in episodic memory function, the primary clinical manifestation of aMCI. For example, dentate gyrus/cornu ammonis 3 is crucial for memory encoding (Eldridge et al. 2005), whereas the subiculum is important for memory retrieval (Eldridge et al. 2005) and scene processing and discrimination (Hodgetts et al. 2017). Therefore, it is really important for us to explore whether and how acupuncture regulates the subregion of the hippocampus.

Overall, we hypothesized that patients with aMCI would show altered hippocampal subregion patterns after acupuncture. Consequently, we aimed to explore the neuroplasticity of acupuncture on the hippocampal subregion of aMCI using a randomized, placebo‐controlled study design, which could enhance our understanding of the neuropathological mechanisms underlying acupuncture in aMCI.

## 2 Materials and Methods

### 2.1 Ethical Statement

The study was meticulously conducted in accordance with the principles outlined in the Declaration of Helsinki and adhered to Good Clinical Practice guidelines. It received approval from the local ethics committees of Shenzhen Hospital of Traditional Chinese Medicine (K2023‐117‐02). Prior to their participation, all individuals involved provided their written informed consent, ensuring a transparent and ethical research process.

### 2.2 Study Design

The study was conducted at the acupuncture department of Shenzhen Traditional Chinese Medicine Hospital in China, spanning a period from October 2023 to November 2024.

This was a prospective, randomized, controlled clinical trial. Patients with aMCI were randomly assigned to the VA or SA in a 1:1 ratio, utilizing an envelope lottery method for assignment. The randomization process utilized sequentially numbered, opaque, sealed envelopes prepared by an independent statistician. Each enrolled participant received the next consecutively numbered envelope containing group assignment. Envelopes were opened only after baseline data collection, ensuring allocation concealment.

Acupuncturists were not blinded to group allocation due to the inherent nature of acupuncture interventions. The patients, evaluators and statisticians were kept blinded throughout the research and data analysis process, ensuring objectivity and minimizing bias in the study's outcomes.

### 2.3 Participants

The eligible participants were Han nationals, right‐handed, who met the following inclusion and exclusion criteria:

The inclusion criteria for patients with aMCI were as follows: (1) diagnosed based on the aMCI diagnostic criteria proposed by Petersen (Petersen 2004); (2) age range between 50 to 75 years; (3) objective evidence of memory impairment, defined as an AVLT Fifth Trial score more than 1.5 standard deviations (SD) below the age‐adjusted normative mean, corroborated by a MMSE score between 24 and 28, and a MoCA score between 19 and 26; and (4) self‐reported memory impairment or insider complaints, with a disease course of > 6 months.

The exclusion criteria were as follows: (1) current or past neurological disorders or current neuropsychiatric disorders affecting memory, such as stroke, seizure disorders, major depressive disorder, or schizophrenia; (2) history of acupuncture treatment or other cognitive function‐related treatments within the past month; (3) inability to undergo clinical evaluations due to severe hearing impairment, aphasia, visual impairment, or contraindications for magnetic resonance imaging (MRI) scans; (4) unstable chronic conditions affecting the heart, kidneys, lungs, liver, or other organs; (5) systemic diseases that could potentially induce cognitive impairment, including epilepsy, Parkinson's disease, severe anemia, anthrax, HIV, alcohol and drug addiction, syphilis, or thyroid dysfunction.

**FIGURE 1:**
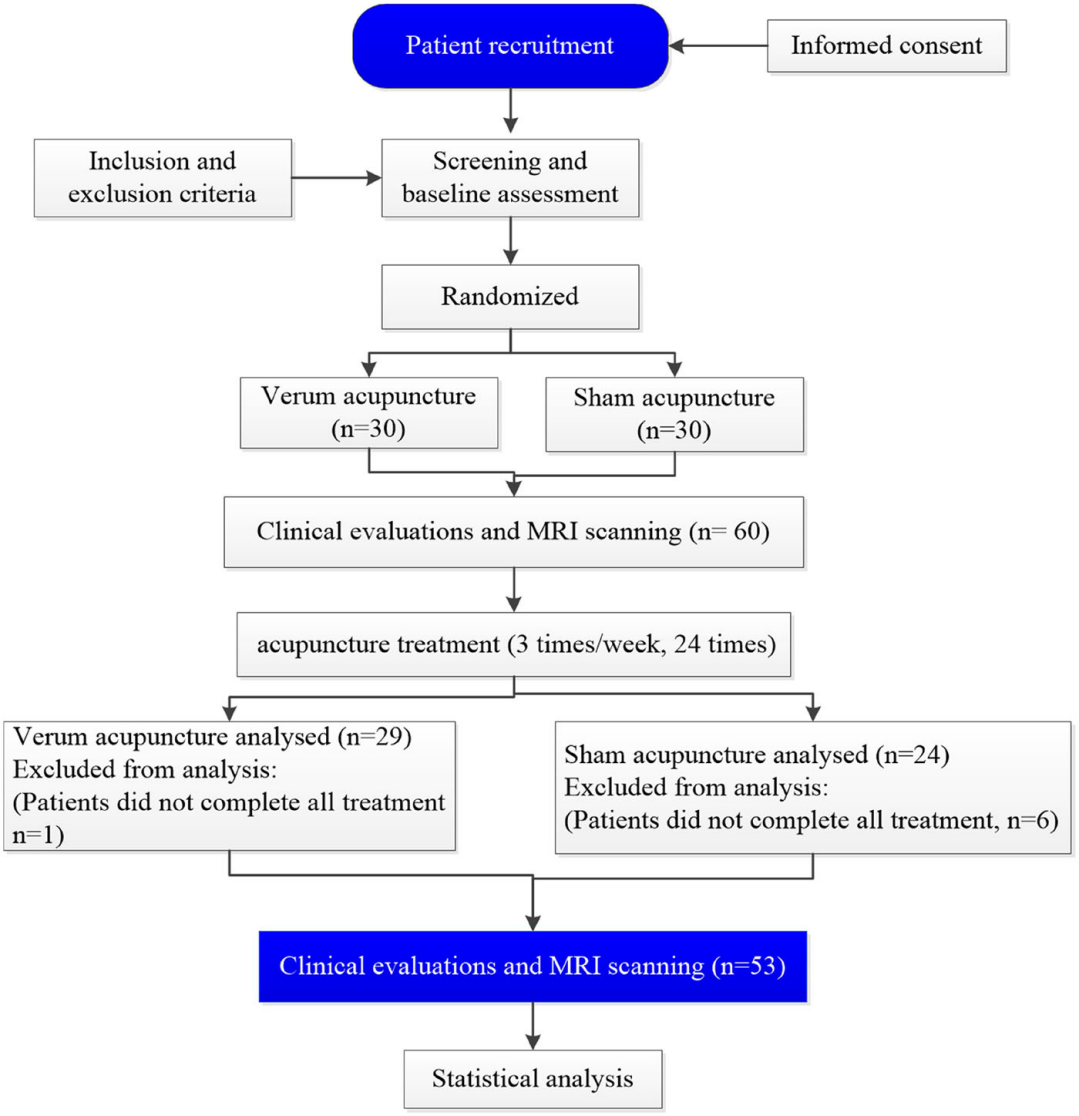
The study flowchart.

Following the initial screening for eligibility, 60 patients with aMCI met the inclusion criteria. However, seven patients were subsequently excluded due to non‐adherence to the treatment protocol. Consequently, 29 aMCI patients were treated with VA and 24 were treated with SA (Figure 1 and Table 1).

**TABLE 1:** Participant characteristics.

Characteristics	Verum acupuncture			Sham acupuncture			*p*
Numbers	29			24			—
Age (mean ± SD)	59.03 ± 6.84			61.71 ± 7.5			0.181^a^
Gender (female:male)	21:8			16:8			0.206^b^
Education (mean ± SD)	12.59 ± 2.08			11.13 ± 3.60			1.461^a^
	Before treatment	After treatment	^C^	Before treatment	After treatment	^C^	0.348^d^
MMSE (mean ± SD)	26.31 ± 1.491	28.97 ± 0.944	< 0.001	26.67 ± 1.579	27.96 ± 1.681	0.001	0.005^e^
MoCA	22.69 ± 2.14	27.1 ± 1.698	< 0.001	22.79 ± 2.553	24.21 ± 2.284	0.027	< 0.001
AVLT_immediate recall	15.93 ± 3.595	24.69 ± 4.327	< 0.001	15.17 ± 3.655	21.08 ± 4.596	0.003	0.035
AVLT_N4	4.76 ± 2.166	8.55± 2.308	< 0.001	4.71 ± 1.781	7.71 ± 1.876	0	0.184
AVLT_N5	4.21 ± 2.32	8.07 ± 2.645	< 0.001	3.92 ± 1.932	7.25 ± 1.962	0	0.392
AVLT_N6	4.38 ± 2.456	8.31 ± 2.509	< 0.001	3.96 ± 1.944	7.17 ± 2.426	0	0.337
AVLT_N7	20.21 ± 3.256	22.86 ± 1.302	< 0.001	26.31 ± 1.491	28.97 ± 0.944	0.127	0.056

Abbreviations: AVLT, auditory verbal learning test; MMSE, mini‐mental State Examination; MoCA, Montreal cognitive assessment; SD, standard deviation.

^a^ represents two sample *t*‐tests.

^b^ represents *χ* ^2^ test.

^c^ represents paired *t*‐tests.

^d^ represents two sample *t*‐tests of MoCA scores between sham and verum groups before treatment.

^e^ represents two sample *t*‐tests of MoCA scores between sham and verum groups after treatment.

### 2.4 Clinical Evaluation

Patients underwent comprehensive clinical assessments and MRI scans at two critical points: prior to the commencement of acupuncture treatment and within 72 h after the final session. Cognitive function was rigorously evaluated using the MMSE and the MoCA. In addition, episodic memory was meticulously assessed through the Chinese Auditory Verbal Learning Test (AVLT‐H). This test encompasses several key components, including: immediate recall measures (AVLT_N1‐N3), a 5‐minute delayed recall (AVLT_N4), a 20 min delayed recall (AVLT_N5), cued recall (AVLT_N6), and a recognition test (AVLT_N7).

These assessments were designed to provide a thorough evaluation of the patients' cognitive and memory capabilities both before and after the acupuncture intervention.

### 2.5 Acupuncture Treatment

**FIGURE 2:**
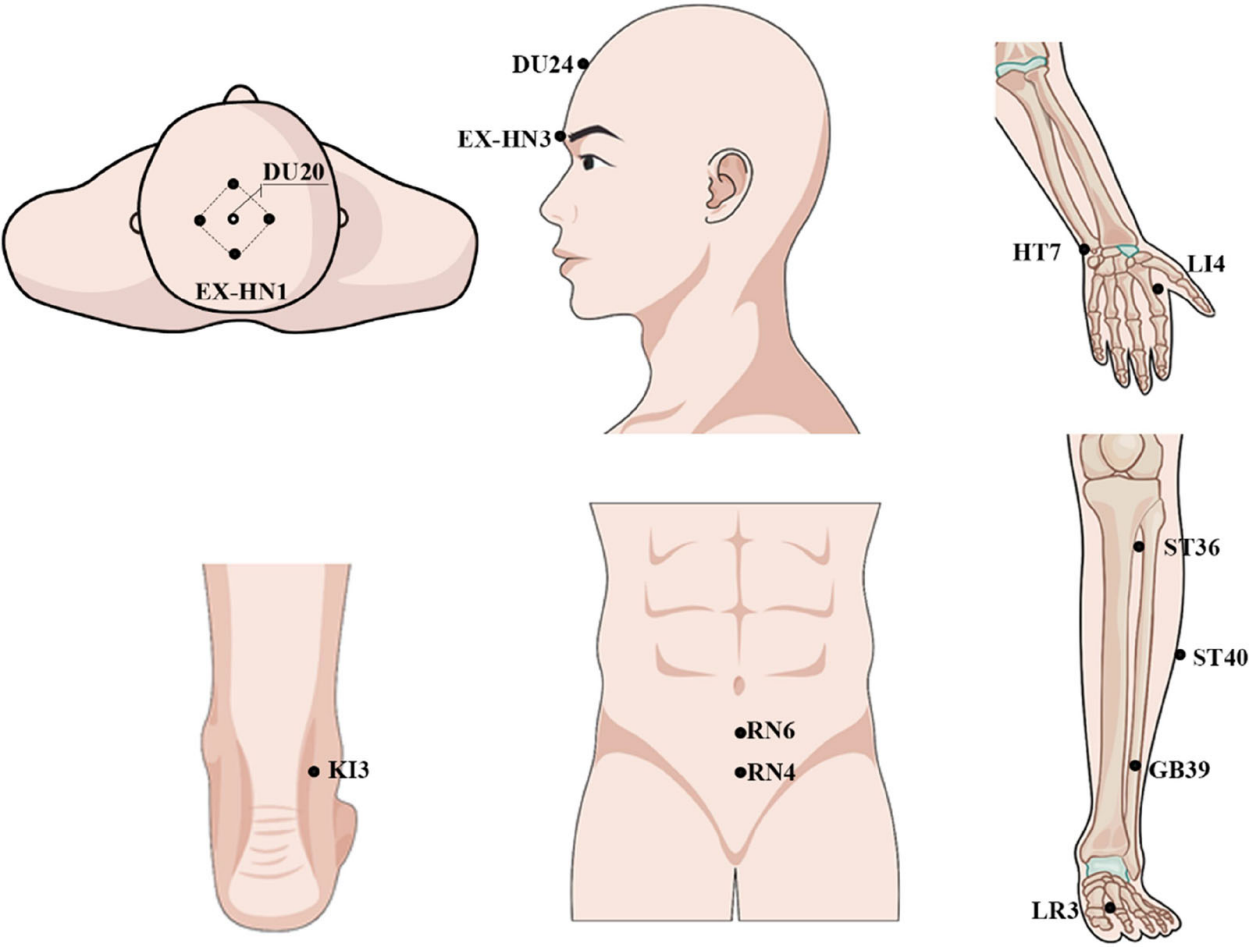
Location of verum acupoints.

All patients with aMCI received 24 sessions (three times a week for eight weeks) of 30‐min VA or SA treatments. The treatments were either VA or SA. Skilled acupuncturists performed manual acupuncture at 20 designated acupoints (DU20, DU24, RN4, RN6, EX‐HN1, EX‐HN3, bilateral LI4, HT7, ST36, ST40, GB39, KI3, and LR3) (Figure 2), adhering to the traditional methods and protocols to ensure the efficacy and consistency of the treatment.

Patients in the VA group received treatment with VA needles inserted at specific acupoints. Table S1 lists details of the acupoints used in the VA. The needles were manipulated by lifting, pulling, twisting, or turning to achieve Deqi (a sensation that includes soreness, numbness, distention, and heaviness). The treatment lasted 30 min, and during the needle retention period, the needles were manipulated by manual twirling, lifting, and thrusting every 10 min to maintain the Deqi sensation.

Patients in the SA group received superficial puncturing at nonacupoints. Acupuncture needles were inserted at nonacupoints, which were 0.1–0.2 cun near the above 20 obligatory acupoints. The nonacupoints were punctured superficially to a depth of 0.1–0.3 cun using 0.25 mm × 13 mm acupuncture needles. Patients in the SA group also received the same duration of treatments as those in the VA group but without achieving the Deqi sensation. The location of the nonacupoints is displayed in Table S2.

### 2.6 MRI Data Acquisition

All patients underwent two MRI scans: 12–24 h before the first acupuncture and 24–72 h after the last acupuncture. The 3D‐T1 MRI scans were conducted with a 3.0 T MRI scanner (Siemens MAGNETOM Prisma 3.0 T). The parameters for the T1 images were as follows: repetition time (TR) = 2200 ms, echo time (TE) = 2.45 ms, flip angle = 8°, field of view = 256 × 256 mm^2^, matrix size = 256 × 256, voxel size = 1 × 1 × 1 mm^3^, and slices = 175.

### 2.7 Data Processing

All T1 images were processed using the standard longitudinal segmentation pipeline provided in Freesurfer 7.1.1 (https://surfer.nmr.mgh.harvard.edu/fswiki/rel7downloads). First, data from both time points were processed longitudinally to generate an unbiased within‐subject template. The remaining steps included motion correction, intensity normalization, cranial stripping, conversion to the Montreal Neurosciences Institute template, segmentation, creation of cortical surfaces, and segmentation. Finally, we obtained hippocampal subregion volumes and estimated the total intracranial volume (eTIV). The hippocampus was automatically segmented into 19 subregions in each hemisphere, including the parasubiculum, fimbria, hippocampal fissure, hippocampal tail, presubiculum head, presubiculum body, subiculum head, subiculum body, the cambium (CA)1 head, the CA1 body, the CA3 head, the CA3 body, the CA4 head, the CA4 body, the granule cell molecular layer of the dentate gyrus (GC‐ML‐DG) head, the GC‐ML‐DG‐body, the molecular layer hippocampus head, the molecular layer hippocampus body, and hippocampus‐amygdala‐transitional area (HATA) (Xu et al. 2023). All volumetric analyses included adjusting for age, sex, education, and estimated eTIV as covariates.

**FIGURE 3:**
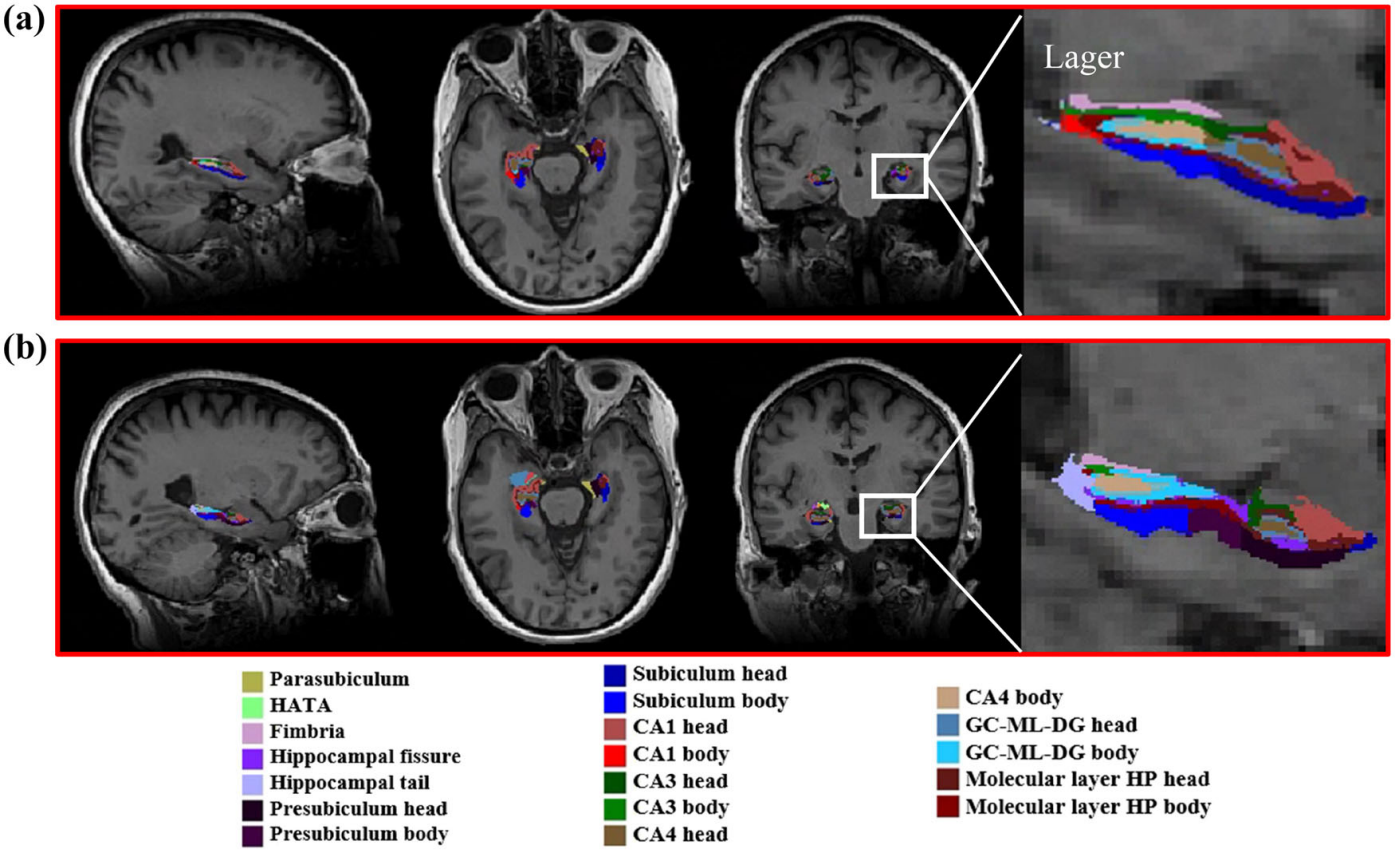
Segmentation results of hippocampus on T1 MRI scans of an amnestic mild cognitive impairment (aMCI) patient before and after acupuncture. (a) 19 hippocampal sub‐regions before verum acupuncture, and (b) 19 hippocampal sub‐regions after verum acupuncture. Abbreviations: CA, cornu ammonis; GC‐ML‐DG, granule cell molecular layer of dentate gyrus; HATA, hippocampus–amygdala transition‐area; and HP, hippocampus.

The automated segmentation of the hippocampus in one of the patients is displayed in Figure 3.

### 2.8 Statistical Analyses

Chi‐square analysis was employed to examine gender differences, and an independent group *t*‐test was utilized to examine inter‐group differences in demographic and clinical scales between VA and SA.

The two‐way ANOVA was deployed to explore the interaction effect between the two groups (VA and SA) and time (before and after treatment) and the main effects of group and time on hippocampal volumes at subregion level.

Then, paired two‐sample t‐tests were performed to explore hippocampal subregion differences before and after acupuncture. Finally, the independent sample *t*‐test was used to examine the change rate ((post‐pre)/pre) of the hippocampal subregion of preintervention and postintervention in VA and SA.

Moreover, correlation analyses between volume changes and changes in scale scores were performed using partial correlation, controlling for age, gender, and education. All statistical analyses were performed using IBM SPSS Statistics version 26; the significance level was set at *p* < 0.05 with two‐tailed.

## 3 Results

### 3.1 Demographic and Clinical Characteristics

No significant differences were observed in the distributions of age, gender, and education between VA and SA groups. MMSE, MoCA, and AVLT‐H scores were increased in VA and SA groups, whereas the VA group showed greater increasementsin MMSE, MoCA, and AVLT_immediate recall scores than the SA group (Table 1).

### 3.2 Longitudinal Volume Changes in Hippocampal Subregions

**FIGURE 4:**
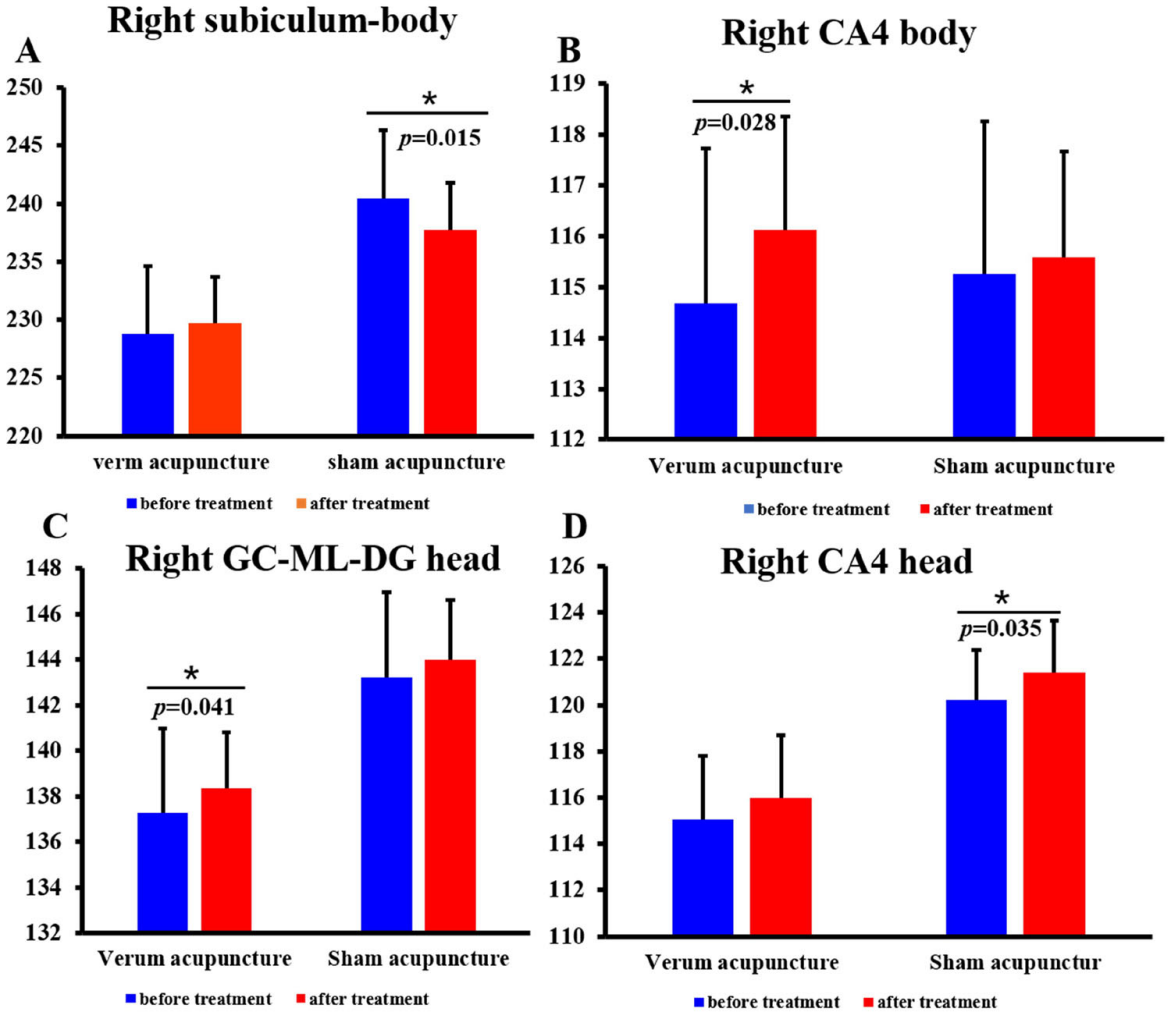
Interaction effects and condition effects of hippocampal subregions in the aMCI after acupuncture. (A), interaction effects; (B–D), condition effects. Brain regions showed significant interaction effects and condition effects of regional homogeneity.

Mixed‐effect analysis showed interaction effects of volume in the right subiculum body (*p* = 0.006). In particular, the volume values significantly decreased in the SA group (*p* = 0.015) but moderately increased in the VA group. No significant interaction effects were observed in other hippocampal subregions (Figure 4).

Condition effects revealed that right GC‐ML‐DG head, right CA4 body, and right CA4‐head exhibited the same trends of change. Specifically, a significantly increased volume in the right GC‐ML‐DG head (*p* = 0.041) and right CA4 body (*p* = 0.028) was observed after VA. However, no significant difference was observed in the SA group. In addition, there was a significant increase in the volume of the right CA4‐head (p = 0.035) after SA, no significant difference was observed in the SA group (Figure 4).

**FIGURE 5:**
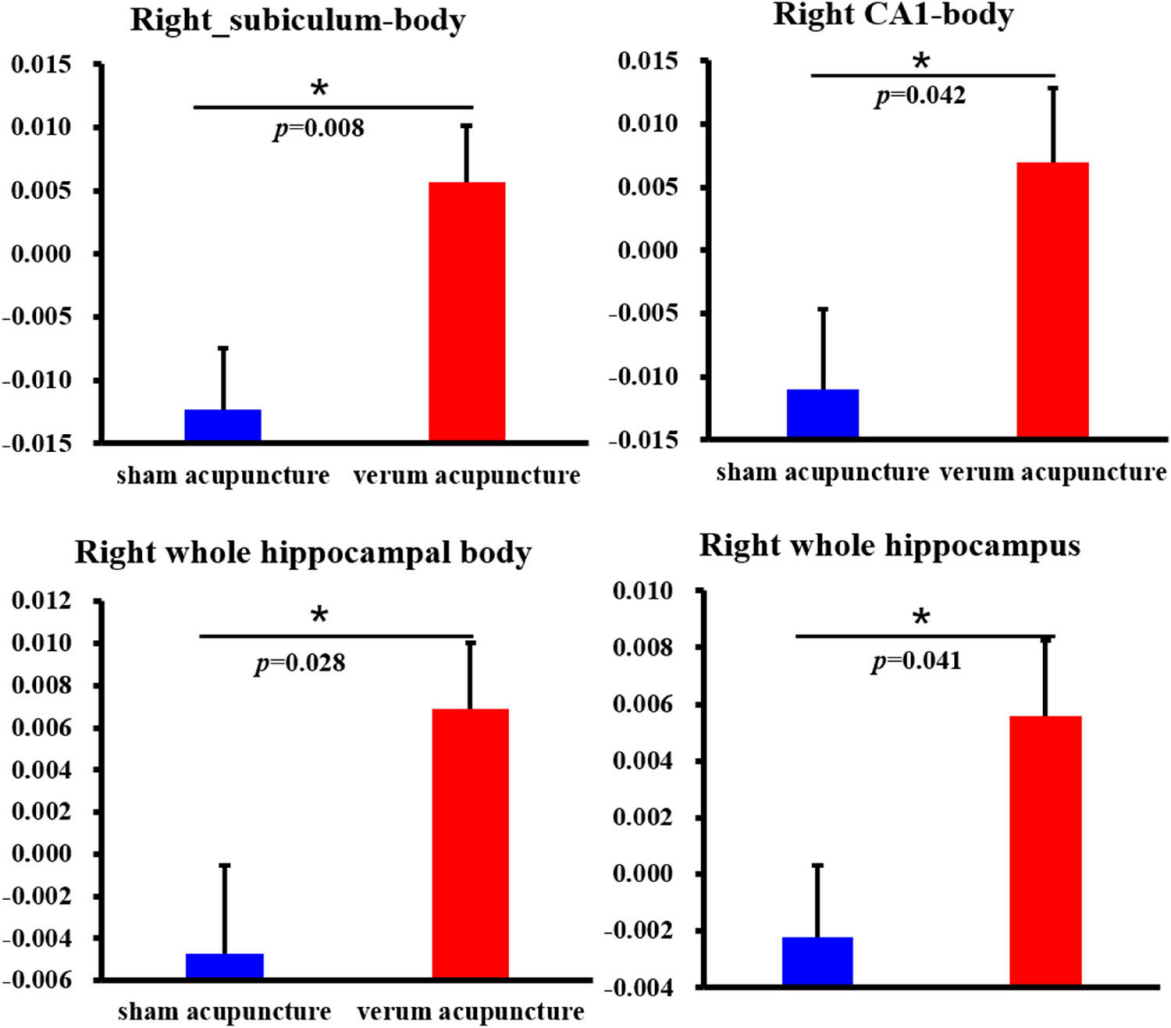
Hippocampal subregions which showed volume difference (after–before) in aMCI between VA and SA group.

When comparing changed values between VA and SA, patients with aMCI showed significantly higher increased volume in the right subiculum body (*p* = 0.008), CA1‐body (*p* = 0.042), whole hippocampus body (*p* = 0.041), and whole hippocampus (*p* = 0.028) in VA than in SA. Notably, the volume of the above hippocampal subregion increased following VA but decreased following SA (Figure 5).

### 3.3 Correlation Between Longitudinal Changes and Clinical Effects

**FIGURE 6:**
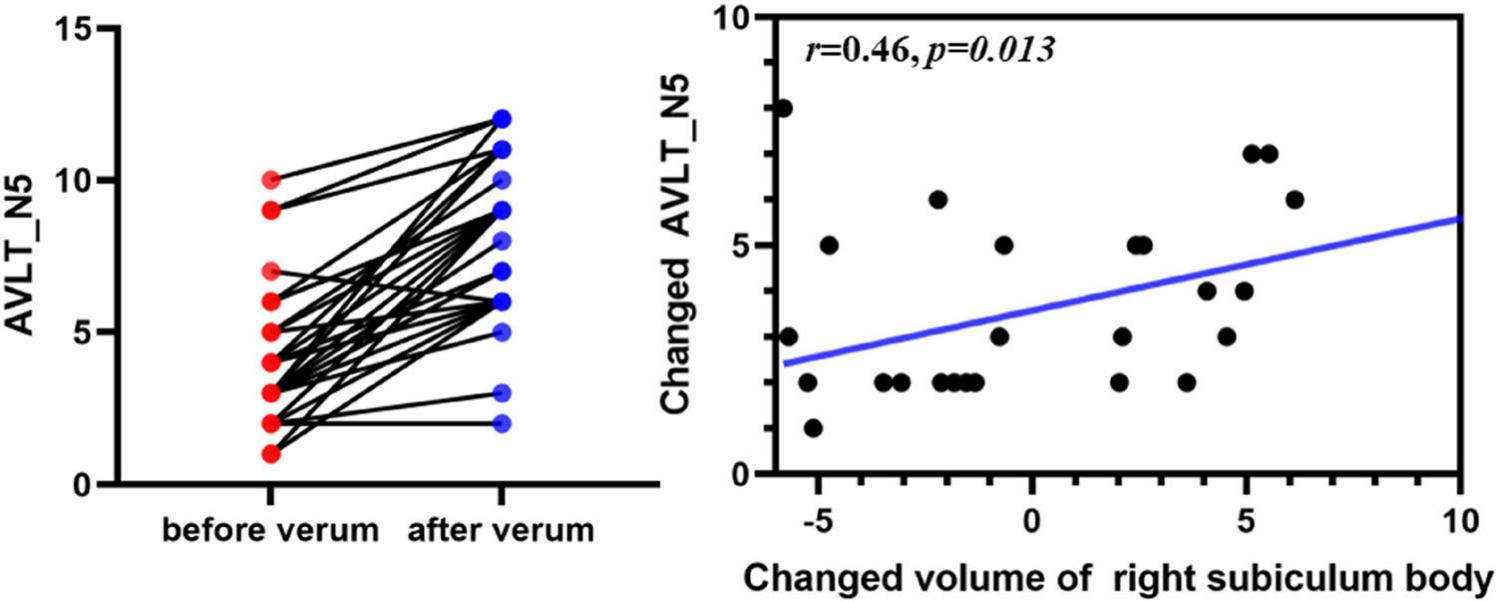
Relationship between changes of right subiculum volume and clinical measure in VA group.

A significant correlation was found between the increased volume of the right subiculum body and improved AVLT_N5 in VA (*p* = 0.013) (Figure 6).

## 4 Discussion

We found that acupuncture could not only improve memory and cognitive function but could also increase the volume of the right subiculum body, right GC‐ML‐DG head, right CA4 body, CA1 body, whole hippocampus body, and whole hippocampus in aMCI. Moreover, a significant correlation was found between the increased volume of the right subiculum body and improved AVLT_N5 in VA. Acupuncture can improve the clinical symptoms and remodel the hippocampal structure of aMCI. In the mechanism of Chinese medicine theory, the disease of aMCI is located in the brain, and it is closely related to the kidney, the spleen, and the heart, and the deficiency of the brain marrow will lead to the loss of nourishment of the Yuan Shen, and the deficiency of qi and dampness obstruction over a long period of time, and the Yangqi will not be uplifted, and then acupuncture can achieve the effect of regulating qi and resolving phlegm and filling in the medulla oblongata, which will then improve the cognitive symptoms and the structure of hippocampus Re‐plasticization.

We found that acupuncture can improve the memory and cognitive function of patients with aMCI, paralleling the findings of previous studies (Deng and Wang 2016; Lai et al. 2020). Besides, we found that both VA and SA could improve cognition and memory function, which aligns with a previous study (Choi et al. 2021). Thus, we compared the changed difference between VA and SA and found that VA led to greater improvements in cognitive and memory function than SA in MMSE, MoCA, and AVLT_immediate recall. The efficacy of SA has been discussed and analyzed in our previous study (Zhang et al. 2022). It is a common phenomenon that SA can also produce clinical results (Fan et al. 2022; Tu et al. 2021). As highlighted by one study, equating the use of SA with the use of placebo drugs in pharmaceutical research is unreasonable because SA can produce both specific and nonspecific effects (Kim et al. 2022). In addition, an eight‐week treatment course was too short to distinguish the difference in efficacy between VA and SA. Acupuncture has a dose‐dependent effect. Tu et al. found no statistical significance between the VA and SA groups for knee osteoarthritis. Interestingly, by Week 16, the VA group also showed benefits in pain and function in knee osteoarthritis compared with the SA group (Tu et al. 2021). However, further long treatment sessions, and follow‐up are necessary in future studies.

To our knowledge, this is the pioneering study to delve into the structural plasticity of the hippocampal subregion in response to acupuncture treatment for aMCI. While existing research has indicated that acupuncture can modulate the functional activities of the hippocampus in individuals with aMCI (Wang et al. 2012; Chen et al. 2013), few studies have demonstrated the structural alterations of acupuncture for aMCI. The reasons are: (1) it is challenging for acupuncture therapy to modulate the morphology of the aMCI, such as voxel‐based morphometry; and (2) acupuncture may modulate more microscopic volumes, such as subregions, which require further investigation. Interestingly, our study verifies the second hypothesis: acupuncture can increase the hippocampal subregion volume of aMCI. This suggests that hippocampal subregions are remodelable, might indicating that progression to AD can be slowed down.

Although it is surprising that an increase in hippocampal subfield volumes was reported following only eight weeks of acupuncture treatment in our study. In fact, a recent study (Wang et al. 2024), which demonstrated that acupuncture significantly increased hippocampal volume in patients with Subjective Cognitive Decline (SCD)‐ an early stage of aMCI, is in line with us. Critically, this study employed an identical treatment protocol. Moreover, we also found that many previous studies have revealed that acupuncture can regulate hippocampal structure by promoting the release of neurotrophic factors such as BDNF, regulating neuroinflammation (Zhang et al. 2015; Shen et al. 2024), improving cerebral blood perfusion (Cao et al. 2021), enhancing synaptic plasticity (Li et al. 2012), and affecting changes in glial cells (Luo et al. 2017). Finally, analogous non‐pharmacological interventions, such as Baduanjin (Wan et al. 2022) and aerobic exercise (ten Brinke et al. 2015), have also demonstrated the capacity to reshape hippocampal subregion volumes in cognitively impaired populations, reinforcing the plausibility of structural plasticity within the hippocampus.

Here, interaction effects on the volume of the right subiculum body of acupuncture for aMCI were found. In particular, the volume decreased significantly in the SA group but increased moderately in the VA group. Consistent with our results, recent research revealed that resistance exercise slowed posttraining CA1, subiculum, and dentate atrophy (Broadhouse et al. 2020). Notably, although the hippocampal subregion of acupuncture for aMCI has been poorly investigated, the importance of the hippocampal subregion in the pathogenesis of aMCI has been highlighted. The subiculum is central to the “connected hippocampus,” yet its significance is often overlooked. Previous studies found that the highest rate of reduction in aMCI is the CA1 and subiculum regions (Madusanka et al. 2019); the faster volume loss in the subiculum suggests a higher risk of disease progression in patients with MCI (Zeng et al. 2021). Additionally, the hippocampal subiculum is associated with Aβ/tau markers in the clinically defined stages of AD (Wu et al. 2021) and is crucial in memory, visual cognition, and emotion (Chase et al. 2015). A recent study revealed that subiculum lesions are sufficient to impair spatial memory tasks, with greater sensitivity for tests of spatial working memory than reference memory (Aggleton and Christiansen 2015), further supporting the conclusion that acupuncture improves episodic memory by increasing subiculum volume. Furthermore, no significant difference in the VA group was found, possibly due to the small sample size and insufficiently long treatment course. Accordingly, future studies are necessary to verify our results with larger samples and longer treatment durations.

In this study, patients with aMCI showed significantly increased volume in the GC‐ML‐DG head, right CA4 body, and right CA4 head following VA, while these changes were not observed with SA. Previous research found that GC‐ML‐DG could be employed as neuroimaging biomarkers to assist in the clinical diagnosis of MCI and AD (Qu et al. 2023). Atrophy of the bilateral CA4 and subiculum subfields was higher in AD than in MCI and HC (Madusanka et al. 2019). Moreover, the subiculum and CA4/DG are associated with objective memory performance and informant reports of memory function (O'Shea et al. 2022). The fact that VA and SA work in the same hippocampal subregion suggests that SA is not entirely inactive and can produce some effects. In addition, patients with aMCI showed significantly increased volume in the right CA4‐head following SA, while VA did not show this effect, indicating the possible comforting impact of acupuncture. After all, the SA group also showed some improvements in episodic memory and cognitive function.

Furthermore, patients with aMCI showed significantly increased volume in the right subiculum body, right CA1 body, whole hippocampus body, and whole hippocampus in the changed value in VA as compared with SA. The volume of the above hippocampal subregion increased following VA but decreased following SA, suggesting the specificity of VA for aMCI. The hippocampal CA1, a major component of the medial temporal lobe memory circuit, is a crucial region for memory encoding and formation (Ginsberg et al. 2019). Moreover, a study suggested that the hippocampal CA1 subfield predicts episodic memory impairment in Parkinson's disease (La et al. 2019). The aMCI of this study was an early MCI, as described in previous studies, which showed hippocampal surface changes mainly in the CA1 region and ventral subiculum (Lee et al. 2017). Overall, we infered that the above hippocampal subregion, closely related to cognition and memory function, contributes to memory impairment in aMCI; remodeling of the hippocampus subregion of acupuncture for aMCI is crucial for improving cognition and memory.

Interestingly, the remodeling effect of acupuncture on the hippocampal subregion was all on the right side, which aligns with a previous study that the right hippocampus was more atrophic than its left counterpart in MCI (Minkova et al. 2017). Finally, we found that VA prevented atrophy in the whole hippocampus. These results suggest that acupuncture can reverse the atrophy of the hippocampal subregion and provide a basis for acupuncture efficacy prediction and individualized treatment. In the future, larger sample sizes and longer treatment times need to be implemented to further confirm the therapeutic mechanism of acupuncture. Moreover, with extended treatment time, the acupuncture response to the hippocampal subregion of aMCI will be more definite and meaningful.

Notably, only a significant correlation was found between the increased volume of the right subiculum body and increased AVLT_N5. Consistent with our study, one study revealed that the subiculum volume correlates with AVLT (Zhao et al. 2019). AVLT‐N5 is used for evaluating immediate recall; the subiculum is active during the recollection of an episode (Eldridge et al. 2005) and is associated with recall, such as rapid memory updating and retrieval (Roy et al. 2017). We must acknowledge that our results presented low correlations between alterations in hippocampal subregions and changes in clinical scales, possibly due to the small sample size and insufficient treatment. In summary, we infer that the subiculum may be the target of acupuncture for aMCI, laying the foundation for the mechanism of efficacy and individualized acupuncture therapy for aMCI.

Several limitations of this study must be acknowledged. First, the sample size was small, which may lead to sampling bias. In addition, the treatment course was shorter than desired, which could have contributed to results that differed from expectations. Thirdly, the chosen measure of hippocampal subfields may be considered a limitation when generalizing to other studies that have used different automatic segmentation tools for hippocampal subfields. Then, an 8‐week treatment course may not be sufficient. Currently, we are conducting a study with a 12‐week course and 36 acupuncture sessions. Finally, the lack of strict correction methods may indeed increase the risk of false positive results, and exploratory results need to be validated in future studies with larger samples. Despite this limitation, larger samples, sufficient treatment duration, and multicenter studies are needed to further verify the acupuncture responses to the hippocampal subregion of aMCI.

## 5 Conclusion

This study suggested that acupuncture could selectively induce structural plasticity of hippocampal subregions associated with cognitive effects in patients with aMCI and provided potential biomarkers (especially the right subiculum body) for effective and timely acupuncture interventions in clinical applications. We believe that these findings could deepen our understanding of the underpinning mechanisms of acupuncture for aMCI and offer a new strategy for its treatment.

## Author Contributions

**Jinhuan Zhang**: conceptualization, data curation, formal analysis, visualization, writing – original draft, methodology, investigation, supervision, project administration, writing – review and editing, resources, validation, software. **Bin Yan**: data curation, visualization, investigation, project administration. **Yongfeng Liu**: methodology, project administration, data curation, formal analysis, visualization, writing – review and editing, resources. **Ting Liu**: conceptualization, data curation, methodology. **Liyu Hu**: conceptualization, data curation, formal analysis, supervision, validation. **Xiaohui Zhan**: conceptualization, data curation. **Xinbei Li**: conceptualization, data curation, formal analysis, investigation, methodology. **Chengcheng Zhu**: conceptualization, writing – review and editing, project administration, supervision, visualization. **Jinping Xu**: writing – review and editing, visualization, data curation, investigation, methodology, software, validation, supervision. **Haibo Yu**: writing – review and editing, conceptualization, supervision, validation, resources, methodology, investigation, visualization, data curation.

## Conflicts of Interest

The authors declare no conflicts of interest.

## Peer Review

The peer review history for this article is available at https://publons.com/publon/10.1002/brb3.70748

## Ethics Statement

The study was submitted to and approved by the Shenzhen Hospital of Traditional Chinese Medicine Evaluation Committee (K2023‐117‐02), China, and was conducted in accordance with the principles stated in the Declaration of Helsinki. Written informed consent was obtained from each patient or their guardian.

## Supporting information



Table S1. Location of acupoints in verum acupuncture.
Table S2. Location of non‐acupoints in sham acupuncture.

## Data Availability

The data that support the findings of this study are available from the corresponding author upon reasonable request.
