# Supplementary material for: Selective Plasticity of Hippocampal Sub‐Regions in the Amnestic Mild Cognitive Impairment After Acupuncture
Source: Brain Behav. 2025 Aug 21;15(8):e70748. doi: 10.1002/brb3.70748 (PMC12370839; doi:10.1002/brb3.70748)
Supplement: Supplementary file 1 — Table S1. Location of acupoints in verum acupuncture. Table S2. Location of non‐acupoints in sham acupuncture. [file BRB3-15-e70748-s001.docx]

**Table S1. Location of acupoints in verum acupuncture.**

| **Acupoints** | **Locations** | **Depth** |
| --- | --- | --- |
| Baihui (DU20) | On the head, 5 cun straight up in the middle of the front hairline. | 0.5-0.8 cun^a^ |
| Sishencong (EX-HN1) | On the head, 1 cun on each side of the front and back of Baihui | 0.5-0.8 cun |
| Shenting (DU24) | On the head, 0.5 cun straight up in the middle of the front hairline. | 0.5-0.8 cun |
| Yintang (EX-HN3) | At the forehead, In the depression in the middle of the medial end of the eyebrows | 0.3-0.5 cun |
| Qihai (RN6) | In the lower abdomen, 1.5 cun below the umbilical cord, at the anterior midline | 1-1.5 cun |
| Guanyuan (RN4) | In the lower abdomen, 3 cun below the umbilical cord, at the anterior midline. | 1-1.5 cun |
| Shenmen (HT7) | In the anterior carpal region, the ulnar end of the distal metacarpal striation and the radial margin of the flexor Carpi ulnaris tendon | 0.3-0.5 cun |
| Hegu (LI4) | On the back of the hand, at the radial midpoint of the second metacarpal. | 0.5-1.0 cun |
| Zusanli (ST36) | On the outside of the calf, 3 cun under the ST35, on the line between ST35 and ST41 | 1-2 cun |
| Fenglong (ST40) | On the anterior aspect of the lower leg, 8 cun superior to the external malleolus, lateral to ST38, two finger breadth (middle finger) from the anterior crest of the tibia | 1-1.5 cun |
| Xuanzhong (GB39) | On the lateral leg, 3 cun above the tip of the lateral malleolus, anterior edge of the fibula | 0.5-0.8 cun |
| Taichong (LR3) | In the dorsum of the foot, between the 1st and 2nd metatarsals, in the depression in front of the junction of the base of the metatarsus, or touching the pulse of the artery | 0.5-1.0 cun |
| Taixi (KI3) | In the ankle area, in the depression between the tip of the medial ankle and the Achilles tendon | 0.5-0.8 cun |

^a^1 cun (≈20 mm) is defined as the width of the interphalangeal joint of the participant’s thum^b^.

**Table S2. Location of non-acupoints in sham acupuncture.**

| **Acupoints** | **Locations** | **Depth** |
| --- | --- | --- |
| NA1 | 2cm outwards from the level of DU20 | 0.1 cun |
| NA2 | 0.3cm outwards from the level of EX-HN1, between the bladder meridian and Du meridian | 0.1 cun |
| NA3 | 0.5cm outwards from the level of EX-HN1, between the bladder meridian and Du meridian | 0.1 cun |
| NA4 | 0.5cm outwards from the level of EX-HN3, 45°incline upwards | 0.1 cun |
| NA5 | 1 cm on the right of the RN6, between the kidney meridian and the stomach meridian | 0.1 cun |
| NA6 | 1 cm on the right of the RN4, between the kidney meridian and the stomach meridian | 0.1 cun |
| NA7 | 0.5 cm horizontally on the ulnar side of HT7 | 0.1 cun |
| NA8 | the midpoint of the ulnar side of the first metacarpal | 0.1 cun |
| NA9 | On the outside of the calf, 3 cun under the ST35, on the line between ST35 and ST41 | 0.1 cun |
| NA10 | 1 cm behind the level of the ST40 | 0.1 cun |
| NA11 | 1 cm behind the level of the GB39, between the bladder meridian and gallbladder meridian | 0.1 cun |
| NA12 | 0.3 cm horizontally outwards from the level of LR3, between liver meridian and stomach meridian | 0.1 cun |
| NA13 | In the middle of GB40 and ST41 (between the gallbladder and bladder meridian) | 0.1 cun |

NA, non-acupoints.
